# Explaining the health system in a practical way – the use of a simulation game in medical sociology teaching

**DOI:** 10.3205/zma001639

**Published:** 2023-09-15

**Authors:** Katja Götz

**Affiliations:** 1 University Hospital Schleswig-Holstein, Campus Lübeck, Institute of Family Medicine, Lübeck, Germany

**Keywords:** curriculum, health care system, medical sociology, medical training, simulation game

## Abstract

**Objective:**

A simulation game is a valuable method for conveying teaching content in a practical way. The aim was to design a teaching module for medical sociology on the subject of “The German health care system” which would convey the contents and connections to the students in a practical way using a simulation game.

**Project description:**

In addition to the development of scenarios for the simulation game, role cards for various institutions of the health care system were also produced as a result. The students were given the opportunity beforehand to work on theoretical content regarding the German health care system online (the “flipped classroom method”). In the 90-minute face-to-face event the simulation game was played, followed by a feedback session. The initial impressions of the students were collected.

**Results:**

In the 2022 summer semester, a total of 185 students from the 4^th^ pre-clinical semester took part in the seminar. The students were divided into twelve seminars. One scenario was worked on per seminar. The simulation game contributed to a better understanding of the health care system. The students were generally very satisfied with this type of knowledge transfer and thought that this method might well be integrated into teaching in the future.

**Conclusion:**

Communicating the health care system through a simulation game is evidently suitable for explaining clearly complex issues and presenting the various interests of the individual institutions. In addition, a simulation game stimulates critical debate and can contribute to imparting theoretical content in teaching medical sociology in a practical way.

## Zusammenfassung

**Zielsetzung:**

Eine wertvolle Methode, Lehrinhalte praxisnah zu vermitteln, ist ein Planspiel. Ziel war es, ein Unterrichtsmodul in der Medizinischen Soziologie zum Themengebiet „Das deutsche Gesundheitssystem“ zu gestalten, das den Studierenden praxisnah in einem Planspiel die Inhalte und Zusammenhänge vermittelt.

**Projektbeschreibung:**

Neben der Entwicklung von Szenarien für das Planspiel mündeten verschiedene Institutionen aus dem Gesundheitssystem in Rollenkarten. Im Vorfeld erhielten die Studierenden die Möglichkeit, die theoretischen Inhalte zum deutschen Gesundheitssystem online (Methode Flipped Classroom) zu bearbeiten. In der 90-minütigen Präsenzveranstaltung erfolgte die Durchführung des Planspiels gefolgt von einer Feedbackrunde. Die ersten Erfahrungen der Studierenden wurden erhoben.

**Ergebnisse:**

Im Sommersemester 2022 nahmen insgesamt 185 Studierende aus dem 4. vorklinischem Semester an dem Seminar teil. Die Studierenden verteilten sich auf zwölf Seminare. Pro Seminar erfolgte die Bearbeitung eines Szenarios. Das Planspiel trug zu einem besseren Verständnis des Gesundheitssystems bei. Die Studierenden waren allgemein sehr zufrieden mit dieser Art der Wissensvermittlung und es war für sie vorstellbar, dass diese Methode zukünftig in die Lehre integriert würde.

**Schlussfolgerung:**

Das Gesundheitssystem in Form eines Planspiels zu vermitteln, kann als geeignet angesehen werden, um komplexe Sachverhalte anschaulich zu verdeutlichen sowie die verschiedenen Interessenslagen der einzelnen Institutionen darzustellen. Zudem regt ein Planspiel die kritische Auseinandersetzung an und kann in der medizinsoziologischen Lehre zur praxisnahen Vermittlung theoretischer Inhalte beitragen.

## 1. Introduction

The reform of medical studies, in particular the associated new requirements within the framework of the Z curriculum, make it clear that an even closer dovetailing of theory and practice is desired [1], [2]. So innovative teaching formats are needed more than ever. A simulation game is intended to extend or supplement toolkits currently in use in higher education and is part of constructivist didactics. The slogan “Experience, try out and experiment yourself, always convert into your own constructions of an ideal or material nature and discuss in terms of meaning for one’s own individual interests and motivational and emotional state” [3] in particular seems relevant in advocating the use of simulation games. 

Complex theoretical issues should be conveyed with as much practical relevance as possible. In this way, simulations of decision-making processes can make connections obvious and promote interactive learning. Various areas of competence are promoted by simulation games. In particular, independence, willingness to assume responsibility, teamwork and communication skills are among alongside creativity and flexibility [4]. Games can help to activate cognitive learning processes and are an important tool in medical education [5]. In addition, games are used in medical education to supplement traditional teaching, on the one hand to simulate decision-making processes and on the other hand to enrich teaching [6]. So called “serious games” are often used in medical training, in which simulation training or aspects of patient safety are integrated as game content [7], [8]. The various games which exist can be divided into different categories based on the German Games Archive of the University of Marburg. Simulation games belong to category V which means that the game is associated with a high degree of complexity and communication [6]. 

The Federal Agency for Civic Education has a database with around 300 simulation games on civic education, which are mainly used in schools [9]. Simulation games are increasingly being considered in university teaching, especially in economics courses [10], [11], [12]. However, it currently does not contain a simulation game on the German health care system. Due to the fact that the subject of the German health system has always played a central role in the field of medical sociology at universities [13] and that teaching this content is characterized by a high degree of complexity, converting it into a simulation game seems appropriate. 

The aim was therefore to design and evaluate a course activity on the subject of the German health care system which conveys the content and connections to the students in a practical way using a simulation game.

## 2. Project description

### 2.1. Development

The medical sociology seminar takes place in the fourth pre-clinical semester at the University of Lübeck. It consists of two parts. The first part deals with the German health care system and the second part deals with the topic of digitization and its importance for the doctor-patient relationship [14]. So far, the subject of the German health care system has been taught in small groups in the form of seminars. In order to promote interaction and critical reflection by the students in a targeted manner, the teaching was expanded to include a simulation game. Three sample scenarios were developed for the simulation game (see table 1 (Tab. 1)). The scenarios were chosen to reflect the various health care institutions and out-patient and in-patient settings.

In addition, six role cards were developed, i.e. in each scenario, actors or institutions from the health care system were present and had pre-prepared statements on the individual scenarios. Each person with an assigned role discussed the pros and cons from the perspective of their particular institution. The following six institutions, mostly institutions at the federal level serving as examples, were each transferred to a role card: the Federal Joint Committee, the German Hospital Federation, the German Federal Medical Council, the National Association of Statutory Health Insurance Funds, the National Association of Statutory Health Insurance Physicians and the Federal Ministry of Health. The role cards contained general background information and essential tasks of the institution, its position in the health care system and the point of view on the respective scenario. The role card of the German Hospital Federation for Scenario 3 can be viewed in attachment 1 as an example. The role cards were assigned to the participants by name in advance. There were two to three students per role card. A total of six roles per scenario were represented in the simulation game.

### 2.2. Workflow

Students were given the opportunity beforehand to work on the theoretical content of the German health care system online (the “flipped classroom method”) so that everyone would have the same level of knowledge in the simulation game. For this purpose, the 60-page brochure on the German health care system published by the Federal Ministry of Health was made available, which, in addition to the historical structure of the German health care system, also explains the system of statutory health insurance and the relevant institutions [15]. In the 90-minute face-to-face event, there was a brief introduction to the simulation game, followed by two statements (20 minutes each) based on the task from one of the scenarios and then a feedback session. The following overview shows the workflow of the simulation game (see table 2 (Tab. 2)).

### 2.3. Evaluation

The first impressions of the students were collected after the game using flash feedback and a specially developed questionnaire. This consisted of nine variables. A four-point Likert scale (1=strongly agree to 4=strongly disagree) was used. In addition, the questionnaire was rounded off by an open question to allow students to add anything else they would like to communicate. The data was evaluated descriptively with SPSS 27.0^©^. The free text information was categorized and corresponding anchor examples served as evidence. 

### 2.4. Ethics

Within the scope of the present study, only anonymous data was collected using questionnaires, so that the data cannot be linked to specific persons. Prior to the start, the participants received comprehensive information about the purpose of the study. The study was approved by the independent ethics committee of the University of Lübeck (EK number 22-164).

## 3. Results

### 3.1. Sample description

In the 2022 summer semester a total of 183 students from the 4th pre-clinical semester took part in the seminar Structure and Function of the German Health Care System. 182 students took part in the evaluation of the simulation game. These were divided across twelve seminars. The age range of the students was between 18 and 35 years (M=22.5, SD=3.1). Almost 70% of the participants were female. One scenario was worked on per seminar. 

### 3.2. Evaluation results

Just over half of the students rated the time required for the simulation game as appropriate (53%). Almost half of the students (45%) agreed that the simulation game contributed to a better understanding of the health care system and was fun. The students rated the learning effect and provision of additional resources on Moodle as less high or less helpful for the preparation. Almost 40% of the students could imagine integrating this simulation game into the teaching of future semesters.

**Figure 1:**
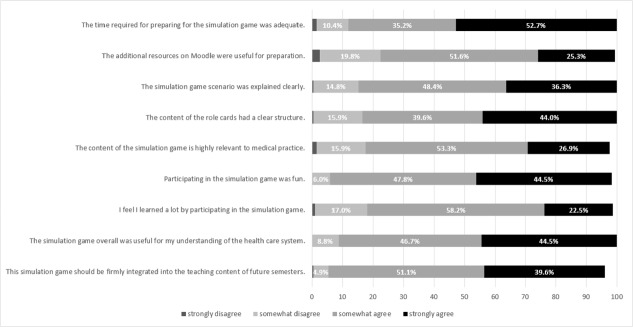
Overview of the evaluation results

Figure 1 (Fig. 1) shows a detailed overview of the distribution of the response frequencies with regard to the variables queried. 

The open question regarding the supplement was answered by a total of 126 students. Some students expressed their general satisfaction with the use of a simulation game for this topic, such as “Thanks, that was fun.” (TN 53) or “Definitely better than dry theory” (TN 128). In addition, the implementation of the simulation game itself was evaluated again by the students in the free text, as the following statement shows, the “time frame for the implementation was well chosen, the role discussions in between were helpful” (TN 2). Furthermore, the added value of this form of teaching was emphasized, as demonstrated by the following statements. “I’m sure that this seminar will stick with you more than talk and chalk” (TN 79) or “finally a different didactic concept” (TN 43). According to the statements of the participants, the simulation game led to clarifying the responsibilities of the individual institutions. “The institutions have become much clearer and the questions/discussions are easy to remember. Definitely keep” (TN 80). In addition to general satisfaction with the implementation of this type of teaching method, the students mentioned various ideas for improving the preparation and the simulation game itself. This was regarding the brochure, which was designed to be read beforehand, and resulted in feedback such as “I would have preferred more material on our role and a shorter overview of the health care system” (TN 171). Likewise, the design of the respective roles and the conduct of the discussion within the framework of the simulation game were seen as needing improvement, as shown by the following statements: “The role card was a bit short. We didn’t have enough arguments at the beginning. It got better in the second round” (TN 95) or “Define the role card more precisely” (TN 31). However, various students also expressed the desire for more background information for preparation: “It would be great if there were more sources for preparation” (TN 22) or “Needed more background information on my organization’s position” (TN 69). 

In the oral flash feedback session after the simulation game had ended, the participants expressed their wish to take on the role of patient representative in order to allow patients to have a say as well. No other suggestions for optimization were mentioned in the direct feedback. The students were generally very satisfied with this type of knowledge transfer. The students also noted that this primarily promoted interaction among each other and strengthened the ability to discuss and engage with the role/institution and its importance in the German health care system.

## 4. Discussion

As the experiences from the simulation game show, it can be a valuable method for teaching the health care system in medical sociology teaching in an interactive way. Various complex topics are explained very clearly and the different interests of the individual actors or institutions can be presented very well. At the same time, the game stimulates critical examination of the various institutions and how they interact within the German health care system and can thus contribute to the practical teaching of theoretical content in medical sociology teaching. It is possible that the importance of medical sociology and, above all, its practical relevance can be better demonstrated in this way, since this is usually only experienced when starting medical work [16]. In addition to the possibility of making teaching more interactive by using a simulation game and thus promoting teamwork and communication skills, the simulation game is also suitable as an online teaching tool. As a result of the COVID-19 pandemic and the associated restrictions, there was a question as to whether face-to-face would resume in the summer semester 2022 and as a result, a didactic method was chosen that conveys complex issues in a practical way and which can be conducted in digital format in small groups. 

The use of the flipped classroom method makes it possible to impart theoretical content in an online self-learning phase. A meta-analysis showed that blended learning has a positive effect on knowledge acquisition in the training of health care professions [17]. The combination of online and face-to-face teaching offers a valuable learning experience in the medical curriculum [18]. 

Based on the experience of running the simulation game for first time, it will be further optimized. The underlying brochure [15] can be supplemented with a knowledge quiz, if necessary, in order to test the relevant learning content in a playful manner. Further background information and sources will be added for the respective role cards. In addition, the role of the Federal Joint Committee will be replaced by the role of the patient representative of the Federal Joint Committee.

After all, the use of such a simulation game can be a valuable addition not only in training but also, for example, in the context of specialization in general practice [19]. The training days of the competence centers for specialization in general practice are based on the Canadian CanMEDS competences [20]. A core competence is the area of management, which also includes knowledge of the German health care system. Thus another area in which simulation games might be worth considering.

For the first time, a simulation game on the German health care system was developed and used in medical sociology teaching. The teaching project was trialed as part of the normal work routines. So far, the game has been carried out at one location, so that only data or statements from one cohort were available. The gender distribution of the participants is roughly comparable to the gender distribution in the subject of human medicine at German universities [21]. Repeating the trial at other locations, e.g. with a larger number of students, could give indications about whether the results can be generalized. 

## 5. Conclusion

With the conception and implementation of the simulation game method, essential content on how the German health care system functions and is structured is conveyed in medico-sociological teaching for the first time. It would also be conceivable to dovetail it with the cross-sectional area “Health economics, health care system, public health”, in which the individual institutions are presented in detail by the respective specialist representatives and, if necessary, discussed on the basis of the scenarios. The next simulation game round for the fourth pre-clinical semester is planned for the summer semester of 2023. This round will include improvements made based on the feedback from the first round. It would also be desirable to conduct this game at other locations and to develop it further in this manner. 

## Competing interests

The author declares that she has no competing interests.

## Supplementary Material

Role card

## Figures and Tables

**Table 1:**
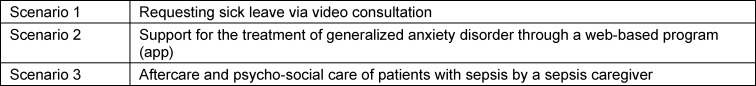
Scenario overview

**Table 2:**
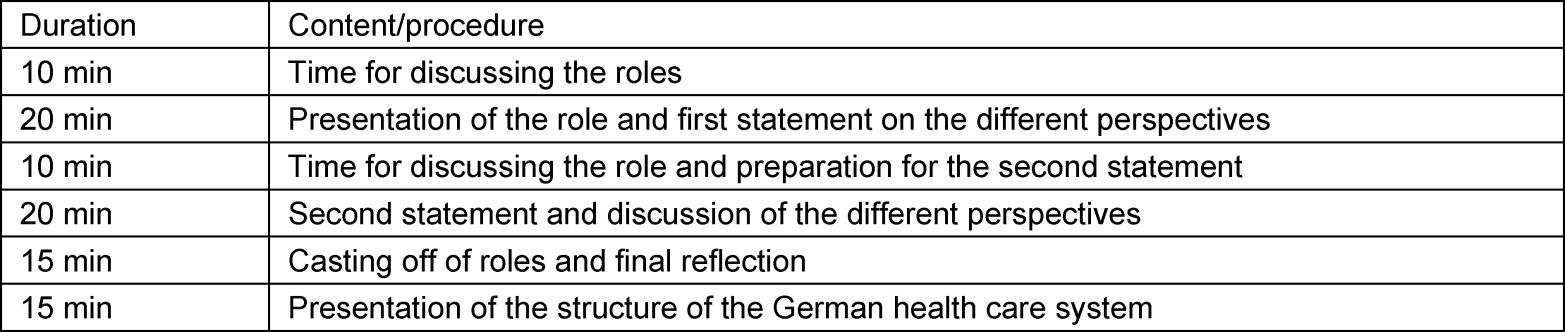
Simulation game or seminar unit workflow

## References

[1] Bundesärztekammer (2021). Reform der Approbationsordnung, ja – Zeitpunkt offen.

[2] Medizinischer Fakultätentag (2021). Struktur des NKLM 2.0 – Teil 3: “Z-Curriculum”.

[3] Reich K, Voß R (1996). Systemisch-konstruktivistische Didaktik. Eine allgemeine Zielbestimmung.

[4] Decker R, Kroll F, Hentschel D, Fortmann LM (2012). Computergestützte Planspiele als Instrument zur Förderung von Soft Skills bei Studierenden. Z Hochschulentwickl.

[5] Akl EA, Pretorius RW, Sackett K, Erdley WS, Bhoopathi PS, Alfarah Z, Schünemann HJ (2010). The effect of educational games on medical students' learning outcomes: a systematic review: BEME Guide No 14. Med Teach.

[6] Bochennek K, Wittekindt B, Zimmermann SY, Klingebiel T (2007). More than mere games: a review of card and board games for medical education. Med Teach.

[7] Dankbaar ME, Richters O, Kalkman CJ, Prins G, Ten Cate OT, van Merrienboer JJ, Schuit SC (2017). Comparative effectiveness of a serious game and an e-module to support patient safety knowledge and awareness. BMC Med Educ.

[8] Gorbanev I, Agudelo-Londoño S, González RA, Cortes A, Pomares A, Delgadillo V, Yepes FJ, Muñoz Ó (2018). A systematic review of serious games in medical education: quality of evidence and pedagogical strategy. Med Educ Online.

[9] Bundeszentrale für politische Bildung Datenbank: Planspiele in der politischen Bildung.

[10] Rubart J, Hartweg E, Schmohl T, Schäffer D, To KA, Eller-Studzinsky B (2019). Planspiele in der Hochschullehre – am Beispiel von Fort Fantastic und ERPsim.

[11] Jacob A, Teuteberg F, Strahringer S, Leyh C (2017). Game-Based Learning, Serious Games, Business Games und Gamification – Lernförderliche Anwendungsszenarien, gewonnene Erkenntnisse und Handlungsempfehlungen.

[12] Kulkarni B, Banerjee R, Raghunathan R (2022). Why students should be taught differently: learner characteristics, learning styles and simulation performance. Simul Gaming.

[13] Dragano N, Deinzer R, von dem Knesebeck O (2018). 3.1. Das deutsche Gesundheitssystem.

[14] Waschkau A, Götz K, Steinhäuser J (2020). Fit for the Future - Entwicklung eines Seminars zu Aspekten der Digitalisierung im Gesundheitswesen als Beitrag der Lehre im Fach Medizinische Soziologie. Z Evid Fortbild Qual Gesundhwes.

[15] Bundesministerium für Gesundheit (2022). Das deutsche Gesundheitssystem.

[16] Stößel U, Weyers S, Götz F, Siegrist J, Stößel U, Trojan A (2022). Entwicklungslinien medizinsoziologischer Lehre an Medizinischen Fakultäten in Deutschland.

[17] Liu Q, Peng W, Zhang F, Hu R, Li Y, Yan W (2016). The effectiveness of blended learning in health professions: systematic review and meta-analysis. J Med Internet Res.

[18] Hege I, Tolks D, Adler M, Härtl A (2020). Blended learning: ten tips on how to implement it into a curriculum in healthcare education. GMS J Med Educ.

[19] Flum E, Maagaard R, Godycki-Cwirko M, Scarborough N, Scherpbier N, Ledig T, Roos M, Steinhäuser J (2015). Assessing family medicine trainees - what can we learn from the European neighbours?. GMS Z Med Ausbild.

[20] Berkenbosch L, Schoenmaker SG, Ahern S, Søjnæs C, Snell L, Scherpbier AJ, Busari JO (2013). Medical residents' perceptions of their competencies and training needs in health care management: an international comparison. BMC Med Educ.

[21] Statistisches Bundesamt (2022). Studierende im Fach Humanmedizin in Deutschland nach Geschlecht bis 2021/2022.

