# Supplementary material for: Explaining the health system in a practical way – the use of a simulation game in medical sociology teaching
Source: GMS J Med Educ. 2023 Sep 15;40(5):Doc57. doi: 10.3205/zma001639 (PMC10594036; doi:10.3205/zma001639)
Supplement: Role card [file JME-40-57-s-001.pdf]

Attachment 1: Role card

DEUTSCHE KRANKENHAUS GESELLSCHAFT DKG

Questions for role preparation:

- Who are the German Hospital Federation (DKG)?
- What is the DKG responsible for?
- How does the DKG appear to the other players in the simulation game?

The German Hospital Federation: As a federal association, the DKG represents 28 member associations of hospital operators: 16 regional associations, 12 umbrella organizations. With this variety of sponsors, the DKG represents the entire breadth of hospital interests.

The DKG supports the hospitals in federal politics. As the umbrella organization of hospital operators, it champions the interests and concerns of hospitals. As the voice of hospitals, the DKG represents hospitals in all health policy decisions, which is why the careful analysis of current health policy and public relations are another central tasks of the DKG. Thanks to the close cooperation with its 28 members, the DKG can prepare and implement legal developments related to hospitals. In addition to its political tasks the DKG has set itself the goals of participating in public dialog, exchanging experience and supporting scientific research. The DKG forms part of self-government in the health care system. Alongside the other partners of self-government – health insurance companies, medical and nursing associations – it decides on all important hospital-related issues. The reimbursement system in the hospital sector and quality assurance in the in-patient sector are largely shaped by the independent DKG. The DKG is a member of the Federal Joint Committee. This makes binding decisions on cross-sectoral issues of out-patient and in-patient care.

More detailed information can be found at: https://www.dkgev.de/

Your tasks: According to estimates by the German Hospital Federation, around 125,000-300,000 people develop sepsis every year. In the case of patients who have survived sepsis or septic shock, there should be follow-up after discharge from hospital with regard to their physical, cognitive and emotional wellbeing. As part of discharge management, it is therefore necessary to keep an eye on aftercare measures. In your opinion, what are the arguments in favor of initiating targeted aftercare measures and which actors should work together more closely?

Attachment 1 to Goetz K. *Explaining the health system in a practical way – the use of a simulation game in medical sociology teaching.* GMS J Med Educ. 2023;40(5):Doc57. DOI: 10.3205/zma001639
